# Supplementary material for: Differences in COVID-19 Vaccine Acceptance, Hesitancy, and Confidence between Healthcare Workers and the General Population in Japan
Source: Vaccines (Basel). 2021 Nov 24;9(12):1389. doi: 10.3390/vaccines9121389 (PMC8707052; doi:10.3390/vaccines9121389)
Supplement: Supplementary file 1 [file vaccines-09-01389-s001.zip › vaccines-1452774-supplementary.pdf]

Table S1. Factors associated with vaccine hesitancy by sex.

		Men (n= 3,395)						Women (n=3,815)					
			hesitant	Crude		Adjusted***			hesitant	Crude		Adjusted***	
		n	n	OR	95%CI	OR	95%CI	n	n	OR	95%CI	OR	95%CI
Age group (years)	20-29	524	95	**1.70**	**1.27-2.27**	**1.45**	**1.00-2.09**	586	126	**1.60**	**1.23-2.07**	**1.38**	**1.01-1.89**
	30-39	564	111	**1.88**	**1.42-2.49**	**1.84**	**1.32-2.56**	748	157	**1.55**	**1.21-1.98**	**1.57**	**1.19-2.08**
	40-49	606	105	**1.61**	**1.22-2.13**	**1.45**	**1.06-2.02**	750	164	**1.63**	**1.28-2.08**	**1.56**	**1.09-2.05**
	50-59	616	91	**1.33**	**1.00-1.78**	1.29	0.94-1.78	658	133	**1.48**	**1.15-1.91**	**1.39**	**1.06-0.83**
	60-69	1,085	125	1.00	reference	1.00	reference	1,073	157	1.00	reference	1.00	reference
State of emergency*		2,144	330	0.97	0.80-1.18	0.99	0.81-1.20	2,453	499	**1.21**	**1.02-1.43**	1.15	0.97-1.38
Population	general	3,090	492	1.00	reference	1.00	reference	3,090	590	1.00	reference	1.00	reference
	Doctor	102	10	0.57	0.30-1.10	0.82	0.41-1.64	18	4	1.21	0.40-3.69	1.71	0.54-5.36
	Nurse	54	8	0.92	0.43-1.96	1.28	0.58-2.84	315	60	1.00	0.74-1.34	1.33	0.95-1.84
	Pharmacist	61	5	0.47	0.19-1.18	0.60	0.23-1.54	74	18	1.36	0.80-2.33	1.72	0.97-3.04
	Therapist	66	9	0.83	0.41-1.70	0.98	0.47-2.06	65	9	0.68	0.34-1.39	0.95	0.45-1.98
	Medical clerk	22	3	0.83	0.25-2.83	0.92	0.26-3.24	253	56	1.21	0.88-1.64	**1.40**	**1.00-1.98**
Mmarital status	Married	2,124	299	**0.75**	**0.62-0.91**	1.14	0.85-1.54	2,377	432	**0.83**	**0.70-0.97**	0.97	0.79-1.19
Have children		1,922	260	**0.71**	**0.59-0.85**	0.94	0.70-1.26	2,264	387	**0.71**	**0.60-0.83**	**0.91**	**0.73-1.112**
Annual house income	≥4 million yen	2,266	328	**0.79**	**0.65-0.96**	0.81	0.66-1.00	2,211	425	0.99	0.84-1.16	0.98	0.82-1.16
Educational level	college or more	2,479	385	1.00	0.81-1.24	1.07	0.86-1.34	2,613	526	1.18	0.99-1.41	1.14	0.94-1.39
Obesity (BMI≥30)		942	146	1.00	0.81-1.23	1.04	0.84-1.30	469	67	**0.66**	**0.51-0.87**	0.71	0.53-0.94
Comorbidity	present	1,237	154	**0.68**	**0.59-0.78**	0.93	0.74-1.17	1,114	209	**0.65**	**0.58-0.73**	**1.24**	**1.02-1.50**
Smoking status	Current smoker	820	100	**0.70**	**0.55-0.88**	**0.63**	**0.50-0.81**	331	62	0.96	0.72-1.28	0.86	0.64-1.17
Havng a history of influenza vaccination													
2020/21season	hesitate	1,755	347	**2.00**	**1.65-2.43**	**1.74**	**1.34-2.31**	1,716	434	**2.01**	**1.70-2.63**	**1.85**	**1.46-2.34**
2019/20season	hesitate	1,871	353	**1.80**	**1.48-2.19**	1.24	0.94-1.65	1,916	454	**1.77**	**1.50-2.09**	**1.26**	**0.99-1.60**
Factors to decide whether to take COVID-19 vaccine													
Age		1,573	196	**0.64**	**0.53-0.78**	0.94	0.73-1.21	1,865	301	**0.67**	**0.61-0.75**	1.100	0.88-1.36
Comorbidity		1,909	236	**0.58**	**0.48-0.70**	**0.76**	**0.60-0.98**	2,407	393	**0.6**	**0.51-0.71**	**0.73**	**0.58-0.91**
Occupation		876	104	**0.67**	**0.53-0.84**	0.94	0.72-1.23	1,416	221	**0.68**	**0.87-0.80**	0.84	0.68-1.05
Vaccination fee		1,034	100	**0.49**	**0.39-0.61**	**0.53**	**0.41-0.69**	1,111	173	**0.70**	**0.58-0.84**	**0.73**	**0.60-0.90**
Doctors' recommendation		604	70	**0.67**	**0.51-0.88**	0.97	0.73-1.30	813	108	**0.58**	**0.46-0.72**	**0.77**	**0.61-0.98**
Vaccine effectiveness		1,935	236	**0.56**	**0.46-0.67**	**0.69**	**0.55-0.88**	2,457	415	**0.65**	**0.56-0.77**	**0.66**	**0.53-0.81**
Duration of vaccine effectiveness		1,261	136	**0.54**	**0.44-0.66**	0.81	0.62-1.04	1,592	256	**0.69**	**0.59-0.82**	**0.85**	**0.69-1.05**
Frequency of adverse event		1,874	265	**0.79**	**0.66-0.95**	**1.42**	**1.13-1.80**	2,504	499	1.12	0.95-1.33	**1.78**	**1.44-2.20**
Families' recommendation		141	11	**0.45**	**0.24-0.84**	0.71	0.37-1.36	182	22	**0.56**	**0.36-0.88**	0.89	0.55-1.44
Epidemic situation of COVID-19		1,035	104	**0.51**	**0.41-0.64**	**0.70**	**0.54-0.90**	1,383	212	**0.66**	**0.55-0.78**	**0.81**	**0.67-0.99**

Emergency statement was evoked in okyo, Chiba, Saitama, Kanagawa, Tochigi, Gifu, Aichi, Kyoto, Osaka, Hyogo, Fukuoka.

**：Adjusted for variables in listed in the Tables.
